# A staged adoption pathway for intraoperative imaging in brain tumor surgery: cost-effectiveness and accessibility in resource-limited neurosurgical settings

**DOI:** 10.3389/fsurg.2026.1865224

**Published:** 2026-07-14

**Authors:** David Zammit Dimech

**Affiliations:** University of Edinburgh, Clinical & Surgical Sciences, Edinburgh, United Kingdom

**Keywords:** 5-aminolevulinic acid, brain tumor surgery, cost-effectiveness, global neurosurgery, health technology assessment, intraoperative imaging, staged adoption pathway

## Abstract

Maximal safe resection extends progression-free and overall survival in glioma surgery, and intraoperative imaging modalities are the means by which this is operationalized. Seven such modalities are now available: 5-aminolevulinic acid (5-ALA) fluorescence, fluorescein sodium, intraoperative magnetic resonance imaging (iMRI), intraoperative ultrasound (iUS), hyperspectral imaging, Raman spectroscopy and stimulated Raman histology, and augmented reality neuronavigation. Their availability is, however, profoundly unequal across high-income and resource-limited neurosurgical settings. This mini review appraises the comparative cost-effectiveness, accessibility, and adoption barriers of these seven modalities, drawing on randomized controlled trials, systematic reviews, formal health-economic analyses, and Lancet Commission data on global neurosurgical capacity. The evidence supports three arguments. That the cost-effectiveness rank order favors 5-ALA, iUS, and fluorescein over iMRI, inverting the apparent technological hierarchy. That accessibility barriers are dominated by capital infrastructure and regulatory status rather than consumable cost. And that no published cost-effectiveness analysis has yet adopted a low- or middle-income country payer perspective. A four-tier staged adoption pathway is proposed, synthesizing the Global IDEAL Sub-Framework, the NASSS framework, WHO-INTEGRATE, and Lancet Commission on Global Surgery indicators, to guide rational adoption sequences for centers operating under capital constraint.

## 1 Introduction

The relationship between extent of resection (EOR) and survival in glioma is one of the best-supported axioms in modern neuro-oncology. Lacroix et al. (1) established in 2001 that resection of at least 98% of contrast-enhancing tumor was an independent predictor of survival in 416 patients with glioblastoma, lengthening median overall survival from 8.8 to 13 months. Sanai et al. (2) subsequently identified a stepwise survival benefit beginning at 78% EOR, and a systematic review and meta-analysis of 41,117 patients across 37 studies confirmed that gross total resection compared to subtotal resection was associated with a relative risk of one-year mortality of 0.62 (number needed to treat: 9) (3). The molecular era has not weakened this relationship but rather, it has refined it. Molinaro et al. (4) showed in a multicenter cohort of 761 patients that maximal resection of both contrast-enhanced (CE) and non-contrast-enhanced (NCE) tumor in patients aged 65 years or younger with isocitrate dehydrogenase (IDH) -wild-type glioblastoma yielded a median overall survival of 37.3 months, approximating the survival of patients with the favorable IDH-mutant subtype. The Response Assessment in Neuro-Oncology (RANO) resect classification has since formalized the dependence of outcome on absolute residual CE and NCE tumor volumes (5), and a 2024 update from the same group confirmed the survival benefit of extensive resection in newly diagnosed glioblastoma without contrast enhancement (6).

Intraoperative imaging modalities are the means by which this resection target is operationalized in the operating room. Seven such modalities are now in clinical or near-clinical use: 5-aminolevulinic acid (5-ALA) fluorescence-guided surgery, fluorescein sodium fluorescence, intraoperative magnetic resonance imaging (iMRI), intraoperative ultrasound (iUS), hyperspectral imaging (HSI), Raman spectroscopy and stimulated Raman histology (SRH), and augmented reality (AR) neuronavigation. Their development trajectories, evidence bases, capital requirements, and regulatory profiles differ substantially.

Their availability differs even more substantially. The Lancet Commission on Global Surgery estimated in 2015 that five billion people lacked access to safe and affordable surgical care (7), and a global modeling study by Dewan et al. placed the unmet annual need for neurosurgical evaluation at 22.6 million cases, with a workforce shortfall of 23,300 neurosurgeons concentrated in low- and middle-income countries (8). The most current global workforce mapping by Gupta et al., published in 2024, estimated 72,967 consultant neurosurgeons worldwide, with densities ranging from 0.12 per 100,000 population in low-income countries to 2.44 per 100,000 in high-income countries. Twenty-nine countries reported no neurosurgeons at all (9). Punchak et al. (10) estimated that only 25.3% of sub-Saharan Africans live within two hours of a neurosurgical center.

These two facts, that resection extent determines survival, and that resection is performed under markedly different infrastructural conditions in different healthcare systems, frame the problem this mini review addresses. The existing literature treats intraoperative imaging as a hierarchy of technological sophistication, with iMRI at the apex. Re-examined under the constraints of finite capital, scarce specialist workforce, and absent regulatory scaffolding that obtain in most of the world's neurosurgical settings, that hierarchy inverts. The article is organized around three arguments. Comparative cost-effectiveness, accessibility, and a proposed staged adoption pathway. This mini review addresses the intraoperative imaging step specifically, not resource-limited neurosurgical practice as a whole. That practice is constrained by far more than imaging technology, including specialist training, equipment maintenance, pathology and molecular diagnostic capacity, and timely access to radiotherapy and chemotherapy, within which the imaging decision is only one link in a broader chain of oncological care.

## 2 The evidence base: A comparative appraisal

### 2.1 5-Aminolevulinic acid fluorescence

5-ALA has the strongest evidence base of any modality. The pivotal phase III randomized controlled trial by Stummer et al. (*n* = 322) reported complete resection in 65% of patients receiving 5-ALA vs. 36% receiving white-light surgery, with six-month progression-free survival of 41% vs. 21% (11). A subsequent *post-hoc* analysis adjusted for selection bias confirmed a median overall survival advantage of 16.7 vs. 11.8 months in patients undergoing complete resection. A 2026 systematic review and meta-analysis by Sanikommu et al. found an unadjusted overall survival hazard ratio of 0.72 favoring 5-ALA in high-grade glioma, although this attenuated to 0.63 and lost statistical significance after adjustment for confounders (12), a methodologically important caveat for downstream economic modeling. Three European cost-effectiveness analyses converge on an incremental cost-effectiveness ratio (ICER) of approximately €9,000 per quality-adjusted life year (QALY) (13–15), and the United Kingdom National Institute for Health and Care Excellence and Ontario Health both endorse 5-ALA as cost-effective at standard willingness-to-pay thresholds (16, 17). The only United States economic analysis identified, presented as the ISPOR 2023 EE396 abstract by Sloan et al., used a cost-per-imaging complete resection rather than cost-per-QALY metric and reported 28%–33% lower cost per imaging complete resection with Gleolan-guided surgery despite the substantial United States vial price (US$2,998 ex-factory, two vials per case) (18). Brazilian neurosurgeons have demonstrated that pharmacy-compounded 5-ALA at approximately US$300 per case is fluorimetrically and clinically equivalent to commercial Gleolan, in a series of 707 patients (19, 20). It is direct evidence that the dominant economic barrier to 5-ALA adoption in jurisdictions where the branded product is unavailable is regulatory rather than pharmacological.

### 2.2 Fluorescein sodium

Fluorescein sodium is a low-cost alternative that exploits blood brain barrier disruption rather than a tumor-specific metabolic pathway. The FLUOGLIO phase II prospective trial reported complete resection in 82.6% of patients with sensitivity 80.8% and specificity 79.1% (21), and a registry of 347 high-grade gliomas demonstrated significant gains in EOR, progression-free survival, and overall survival (22). A direct head-to-head comparison of 5-ALA and fluorescein in 209 patients found no significant difference in extent of resection or overall survival (23), and Eljamel and Mahboob's comparative cost analysis estimated a cost per QALY of US$3,181, the lowest among all modalities with peer-reviewed economic data (24). Fluorescein is approved for ophthalmic angiography but remains off-label for central nervous system tumor use in all jurisdictions.

### 2.3 Intraoperative magnetic resonance imaging

iMRI has the highest capital cost and the most contested evidence base. The Senft randomized controlled trial (*n* = 49, stopped early) reported complete resection in 96% of iMRI patients vs. 68% of controls (25). A 2024 meta-analysis restricted to randomized trials by Wach et al. (3 trials, *n* = 384) reported an odds ratio of 5.40 for gross total resection with iMRI but at the cost of an additional 42 min of operative time (26). The only published full cost-utility analysis, by Abraham et al., reported an ICER of US$76,442/QALY in a United States payer setting and demonstrated that iMRI ceases to be cost-effective at any per-operation cost above US$3,500 (27). Critically, the Roder trial, the only prospective controlled multicenter comparison of iMRI vs. 5-ALA, found no superiority of iMRI for complete resection (81% vs. 78%), progression-free survival, or overall survival, while iMRI added approximately 100 min of operative time (28). A comprehensive three-way comparison by Coburger et al. found that 5-ALA detected 84%, iUS 80%, and iMRI only 50% of histologically confirmed invasive tumor at the resection border (29). These systems differ markedly by field strength. Low-field (<1 T) units offer lower image quality at more modest cost and infrastructure, whereas high-field (≥1.5 T) suites provide superior resolution at substantially greater capital and siting requirements.

### 2.4 Intraoperative ultrasound

iUS has no published randomized controlled trial but a substantial observational evidence base. The Trondheim implementation study reported median overall survival rising from 9.6 to 11.9 months following the introduction of navigated three-dimensional ultrasound (hazard ratio 0.70) (30), and a 2012 population-based natural experiment by Jakola et al. found that early resection guided by three-dimensional ultrasound nearly doubled overall survival in low-grade glioma compared with watchful waiting (31). A 2024 meta-analysis by Pichardo-Rojas et al. (*n* = 732) reported significantly improved overall survival (standardized mean difference 0.26) and a relative risk for gross total resection of 2.02 (32). Contrast-enhanced ultrasound (CEUS) has been shown to be positionally superimposable on gadolinium-enhanced T1 MRI, with a positional discrepancy of less than 2 mm (33). Eljamel and Mahboob estimated a cost per QALY of US$6,049, the lowest among hardware modalities (24). In resource-limited settings iUS is often not merely the cheaper option but the most reliable and sustainable tool available, needing minimal supporting infrastructure, resilient to logistical and supply chain failure, and effective once the operator is trained, making it dependent on training rather than capital. Direct comparisons support this. A cost-effectiveness analysis at a single center found iUS more efficient than iMRI (34), and a histologically controlled study showed linear array iUS approaching high-field iMRI in sensitivity and specificity at the resection margin (35).

### 2.5 Emerging modalities

Three modalities remain at earlier stages of clinical translation. HSI, captured contact-free across multiple wavelengths, has produced promising *in-vivo* benchmarks (macro-F1 approximately 70%) (36) and a 2025 systematic review of preclinical and clinical studies in surgical oncology, including 30 neurosurgery studies, concluded that HSI remains in early technological stages requiring high-quality evidence and multidisciplinary collaboration to support clinical adoption (37, 38). SRH coupled with deep neural networks achieved diagnostic accuracy non-inferior to conventional histology (94.6% vs. 93.9%) in a multicenter prospective trial of 278 cases (39), and the DeepGlioma extension achieved 93.3% molecular classification accuracy in under 90 s (40). The implication for resource-limited settings is potentially leapfrog. SRH coupled with on-platform classifiers could deliver intraoperative molecular diagnosis at centers without on-site neuropathology, a capability previously confined to high-resource referral centers (41). AR neuronavigation has reached clinical accuracy comparable to conventional infrared neuronavigation (target registration error 2.5 mm vs. 2.6 mm) (42), and a tumor-specific systematic review of 22 studies confirmed feasibility but flagged the predominance of low-level evidence (43). None of these three modalities has any published cost-effectiveness analysis.

A network meta-analysis by Naik et al. comparing fluorescein, 5-ALA, and iMRI ranked the modalities by SUCRA (surface under the cumulative ranking curve) for gross total resection. iMRI plus 5-ALA exceeded iMRI alone, which exceeded fluorescein, which exceeded 5-ALA alone, which exceeded white-light control (44). The Cochrane network meta-analysis by Fountain et al., restricted to randomized evidence, reached similar conclusions but graded the underlying evidence quality as low across all comparisons (45). Both reviews highlight a structural feature of the comparative literature. There are no head-to-head RCTs of iMRI vs. iUS, no RCT of iUS in any setting, and the only direct comparison of iMRI vs. 5-ALA at randomized level remains the Roder trial (28).

The seven modalities, their evidence bases, capital and consumable costs, ICERs, and regulatory status are summarized in Table 1.

**Table 1:** Comparative appraisal of intraoperative imaging modalities for brain tumor surgery.

Modality	Best-evidence clinical effect	Capital cost (USD)	Per-case consumable cost (USD)	Best published ICER	Highest evidence level	Regulatory status
5-ALA	Complete resection 65% vs 36% (RCT, *n* = 322) (11)	Microscope filter: $50,000–100,000	$300 (compounded) (19); $2,998/vial branded (18)	€9,000–13,000/QALY (13–15, 17)	Level 1 RCT + multiple SR/MA + HTA	EMA 2007; FDA 2017 (Gleolan); off-label or unavailable in many LMICs
Fluorescein	Complete resection 82.6% (phase II, *n* = 46); sens 80.8%, spec 79.1% (21)	Microscope filter: $50,000–100,000	$5–20/vial	$3,181/QALY (24)	Phase II prospective; observational registries	Off-label for CNS in all jurisdictions
iMRI	Complete resection 96% vs 68% (RCT, *n* = 49) (25); GTR OR 5.40 in 2024 RCT MA (26)	$1–2M (low-field, <1 T) to $5–10M (high-field, ≥1.5 T) installed	None per case	$76,442/QALY (27)	Level 1 RCT + RCT-only MA + CUA	No single-technology HTA by major agencies
iUS	OS SMD 0.26; GTR RR 2.02 (MA, *n* = 732) (32)	Cart system $50,000–200,000; probes $10,000–30,000	None per case	$6,049/QALY (24)	No RCT [per Cochrane NMA (45)]; large observational evidence	Established for non-CNS uses
HSI	Macro-F1 ∼70% (*in-vivo* benchmark) (36); IDEAL stage 1–2a (37, 38)	Research-build six-figure USD; no surgical-grade commercial product	Research-stage	None published	IDEAL stage 1–2a	Not approved as standalone device
Raman/SRH	SRH non-inferior to H&E (94.6% vs 93.9%) (39); DeepGlioma 93.3% molecular accuracy in <90 s (40)	SRH platform ∼$400,000–500,000	None per case (consumables minimal)	None published	IDEAL stage 3–4 (FDA-cleared/CE-marked Invenio NIO)	FDA cleared; CE marked
AR neuronavigation	TRE 2.5 mm vs 2.6 mm conventional infrared (MA) (42)	HMD ∼$3,500; layered on neuronavigation $150,000–400,000	None per case	None published	IDEAL stage 2a–2b	Variable; AR HMDs FDA-cleared

5-ALA, 5-aminolevulinic acid; AR, augmented reality; CE, contrast-enhanced; CUA, cost-utility analysis; EMA, European Medicines Agency; FDA, United States Food and Drug Administration; GTR, gross total resection; H&E, hematoxylin and eosin; HMD, head-mounted display; HSI, hyperspectral imaging; HTA, health technology assessment; ICER, incremental cost-effectiveness ratio; iMRI, intraoperative magnetic resonance imaging; iUS, intraoperative ultrasound; MA, meta-analysis; NMA, network meta-analysis; OR, odds ratio; OS, overall survival; QALY, quality-adjusted life year; RCT, randomized controlled trial; RR, relative risk; SMD, standardized mean difference; SR, systematic review; SRH, stimulated Raman histology; TRE, target registration error.

## 3 Pillar I: cost-effectiveness

The cost-effectiveness landscape is sparse and asymmetric. Of seven modalities, only four, 5-ALA, iMRI, iUS, and fluorescein, have any peer-reviewed cost-effectiveness data. HSI, SRH, and AR neuronavigation have none. This is itself a finding. It means that adoption decisions for the three emerging modalities are being made, where they are made at all, on the basis of clinical effect estimates without economic appraisal.

Among the four modalities with data, the rank order is consistent across European and United States payer perspectives. Fluorescein sits at approximately US$3,181 per QALY (24). 5-ALA sits at approximately €9,000–13,000 per QALY across three independent European analyses (13–15), well below all standard high-income country willingness-to-pay thresholds (€20,000–50,000 per QALY) and within the opportunity-cost-based thresholds derived by Woods et al. for upper middle income settings (46). iUS sits at approximately US$6,049 per QALY (24). iMRI sits at US$76,442 per QALY in a United States setting (27), at the upper bound of conventional cost-effectiveness even in high-income contexts and beyond reach of any plausible LMIC threshold. The Eljamel and Mahboob analysis additionally calculated cost-effectiveness ratios per imaging-complete-resection. US$350 for fluorescein, US$665 for iUS, US$1,784 for 5-ALA, and US$3,625 for iMRI (24), an alternative metric that becomes relevant where direct QALY calculation is infeasible. Reported costs carry two caveats. First, the figures here are denominated in both euros and US dollars, and recent volatility in exchange rates means comparisons across currencies should be read as approximate. Second, drug and technology prices vary substantially between countries and healthcare systems, so the costs cited may not transfer directly to any given setting, as the gap between branded and compounded 5-ALA illustrates.

Three observations sharpen this comparison. First, the only direct prospective controlled comparison between modalities, Roder's iMRI vs. 5-ALA trial across 11 German centers, found no clinical superiority of iMRI on any endpoint (28). The de los Reyes-Nabhan 2025 systematic review concluded explicitly that iMRI is not economically viable for most neurosurgical centers worldwide compared with 5-ALA and intraoperative ultrasound (47). Second, the biological evidence (29) demonstrates that iMRI's poor sensitivity for invasive tumor at the resection border is intrinsic to its reliance on gadolinium enhancement, which by definition does not visualize the non-enhancing infiltrative tumor component whose resection has been shown by Molinaro et al. to extend survival in younger patients (4). Paying more for iMRI buys imaging that systematically fails to reveal the disease component that matters most for the survival outcome that justifies the resection. Third, and this is the most important methodological gap in the literature, no published cost-effectiveness analysis has yet adopted a low- or middle-income country payer perspective. All published analyses use Portuguese, Spanish, United Kingdom, Canadian, or United States viewpoints (13–15, 17, 27). None applies the country-level opportunity-cost thresholds derived by Woods et al., which range from US$3–116 per disability-adjusted life year averted in Malawi to US$4,485–8,018 in Kazakhstan (46). None models catastrophic or impoverishing healthcare expenditure, despite these being core indicators in the Lancet Commission on Global Surgery framework (7).

The Hricak Lancet Oncology Commission on medical imaging and nuclear medicine modeled, across eleven cancers, that scaling diagnostic imaging in LMICs would save 54.92 million life-years between 2020 and 2030 with a return of US$179.19 per US$1 invested (48). The methodological template exists. The brain tumor-specific application has not been built.

## 4 Pillar II: accessibility

Accessibility constraints are not principally about consumable cost. A vial of branded 5-ALA is approximately US$2,998 in the United States (18) and €900–1,200 in the European Union, while compounded preparations at approximately US$300 per case have been validated in Brazil (19, 20). A vial of fluorescein sodium for off-label intracerebral use is US$5–20. These per-case consumable costs are not the rate-limiting barriers to adoption. Equipment cost, moreover, is only one constraint. Sustainable adoption depends equally on trained operators, reliable maintenance and servicing, functioning pathology infrastructure, and the avoidance of treatment delays and discontinuities in oncologic care that can erode any survival gain from improved resection.

The dominant accessibility barriers are four. First, capital cost of platform technology. The microscope filters required for 5-ALA (BLUE 400) and fluorescein (YELLOW 560) fluorescence visualization cost approximately US$50,000–100,000 as an add-on to the operating microscope, often exceeding the per-case dye cost by orders of magnitude over a center's operational lifetime. iMRI installation requires US$1–2 million for low-field systems and US$5–10 million for fully integrated high-field suites. iUS cart-based platforms range US$50,000–200,000 with probes at US$10,000–30,000, requiring no shielding, no cryogens, and no dedicated theater infrastructure. A critical structural advantage. Second, regulatory status. 5-ALA is approved by the European Medicines Agency (since 2007) and the United States Food and Drug Administration (since 2017) for high-grade glioma, but remains unavailable or off-label in many LMIC jurisdictions (49). Fluorescein is approved for ophthalmic angiography but is off-label for CNS tumor visualization in all jurisdictions. iUS, iMRI, HSI, AR, and Raman/SRH modalities have not been the subject of single-technology health technology assessments by any of the major appraisal agencies. The Stimulated Raman histology platform (Invenio NIO) is the exception, having received both FDA clearance and CE marking. Third, trained workforce density. The 2024 global mapping (9) demonstrated profound asymmetry not only in neurosurgeon density but in access to operating microscopes, neuronavigation systems, and imaging infrastructure. iUS in particular is operator-dependent and requires structured training. The international iUS in neurosurgery course described by Mazurek et al., deployed across 12 countries in 2025, offers a template for addressing this barrier (50). Fourth, supporting infrastructure. SRH leverages on-site computational pathology that is unavailable in centers without neuropathology services, and yet this same characteristic makes it potentially leapfrog technology for centers with no neuropathology at all (41). HSI and AR depend on stable broadband for cloud-deployed AI classifiers and on platform integration with existing neuronavigation, both of which are unevenly available. The minimum institutional infrastructure required for each modality is summarized in Table 2.

**Table 2:** Minimum institutional infrastructure required for each modality in resource-limited settings.

Modality	Capital infrastructure	Workforce	Supporting infrastructure	Regulatory or availability barrier
5-ALA	Operating microscope with BLUE 400 filter ($50,000–100,000)	Trained surgeon; pharmacy capability for compounded preparation	Reliable refrigeration for dye; preoperative dosing workflow	Off-label or unapproved in many LMIC jurisdictions
Fluorescein	Operating microscope with YELLOW 560 filter ($50,000–100,000)	Trained surgeon	Standard pharmacy stocking	Off-label for CNS in all jurisdictions
iMRI	Dedicated MRI suite with shielding; $1–10 million installed	MRI technologist; on-call radiologist; trained surgical team	Cryogen supply (high-field); dedicated theater complex; 100 + minutes of additional theater time per case	No single-technology HTA; not feasible at most LMIC institutions
iUS	Cart-based ultrasound with neurosurgical probes ($60,000–230,000 total)	Trained sonographer or surgeon-operator	None beyond standard theater electricity	Established technology, no specific regulatory barrier
HSI	Research-build hyperspectral camera; specialized illumination	Trained operator + AI-classifier maintenance	Computational pipeline for ML inference; broadband for cloud-deployed classifiers	Not approved as standalone surgical device
Raman/SRH	SRH platform (e.g., Invenio NIO) ∼$400,000–500,000	On-site neuropathologist OR AI-classifier-trained operator	Computational infrastructure for classifier inference; broadband for software updates	FDA cleared and CE marked but not widely deployed
AR neuronavigation	AR head-mounted display (∼$3,500) layered on neuronavigation platform ($150,000–400,000)	Trained surgical team familiar with AR overlay workflow	Existing neuronavigation infrastructure; calibrated registration	AR HMDs FDA-cleared; AR-neuronavigation integration variable

AR, augmented reality; HMD, head-mounted display; HSI, hyperspectral imaging; HTA, health technology assessment; iMRI, intraoperative magnetic resonance imaging; iUS, intraoperative ultrasound; LMIC, low- and middle-income country; ML, machine learning; SRH, stimulated Raman histology.

The accessibility argument is reinforced by an analogue from breast cancer surgery. Ward et al. (51) showed that scaling simpler and cheaper imaging modalities, ultrasound and x-ray, captured approximately 70% of survival gains achievable through scaling all imaging in LMICs. The methodological lesson is direct. Prioritizing the most accessible adequate modality, rather than the most sophisticated unattainable modality, is the rational strategy where capital is constrained. Real-world LMIC adoption case studies support this. Kaale et al. (52) described the introduction of iUS at Muhimbili in Tanzania, Moiyadi et al. (53) have built one of the world's most extensive iUS programmes at Tata Memorial in Mumbai, the international iUS in neurosurgery course has trained surgeons across twelve countries (50), and the Duke Division of Global Neurosurgery and Neurology has demonstrated sustainable capacity-building through its long-running partnership with Mulago Hospital in Uganda (54).

## 5 Pillar III: A proposed staged adoption pathway

**Figure 1:**
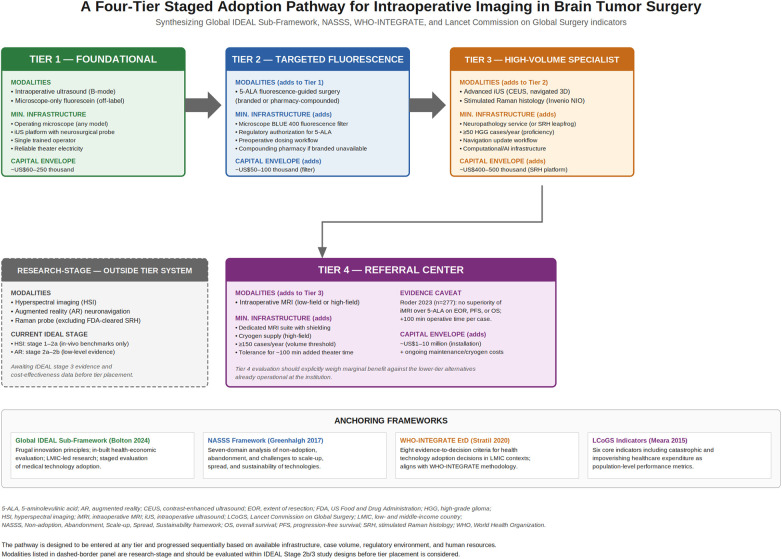
Proposed four-tier staged adoption pathway for intraoperative imaging in brain tumor surgery. Each tier specifies the modality stack, minimum institutional infrastructure, expected extent-of-resection benefit over the tier below, and indicative annual capital envelope. The pathway is designed to be entered at any tier and progressed sequentially. Tier 1 represents the foundational entry point achievable at any center with an operating microscope and reliable electricity. Tier 4 represents the high-volume referral center setting where intraoperative MRI may be added. Hyperspectral imaging, Raman spectroscopy/stimulated Raman histology beyond the FDA-cleared Invenio NIO platform, and augmented reality neuronavigation are positioned outside the tier system as research-stage modalities awaiting IDEAL stage 3 evidence (55, 59). The framework synthesizes the Global IDEAL Sub-Framework (55), the NASSS framework (56), the WHO-INTEGRATE evidence-to-decision framework (57), and Lancet Commission on Global Surgery indicators (7).

The literature contains generic frameworks for surgical innovation adoption, technology diffusion, evidence-to-decision-making, and global surgical capacity, but no neurosurgery-specific staged pathway for intraoperative imaging. This article proposes one, synthesizing four established frameworks. The Global IDEAL Sub-Framework (55), which adapts the Idea-Development-Exploration-Assessment-Long-term study framework for low-resource contexts and emphasizes frugal innovation, in-built health-economic evaluation, and LMIC-led research. The NASSS framework (56), which identifies seven domains explaining technology non-adoption, abandonment, and challenges to scale-up. The WHO-INTEGRATE evidence-to-decision framework (57), which provides eight criteria for health technology decisions in LMIC contexts. The Lancet Commission on Global Surgery indicators (7), which provide population-level performance metrics. The pathway is summarized in Figure 1.

**Tier 1** is the foundational tier. It comprises iUS (B-mode at minimum, with CEUS where available) and microscope-only fluorescein where regulation permits off-label use. The minimum institutional requirement is an operating microscope (any model with adequate magnification), an ultrasound platform with neurosurgical probes, and a single trained operator. Capital outlay is in the tens to low hundreds of thousands of US dollars. The expected EOR gain over no intraoperative imaging is in the range of relative risk 2.0 for gross total resection on the basis of the Pichardo-Rojas meta-analysis (32), with a corresponding overall survival advantage. This tier is achievable in any center with an operating microscope and regular electricity supply.

**Tier 2** adds 5-ALA fluorescence-guided surgery to the Tier 1 platform. The minimum institutional requirement is a microscope filter capable of fluorescence visualization (BLUE 400 or equivalent; capital cost typically US$50,000–100,000), regulatory authorization for 5-ALA use [which may be Gleolan or pharmacy-compounded preparations following the Brazilian model (19, 20)], and a workflow for preoperative dosing and intraoperative protocol adherence. Expected EOR gain over Tier 1 alone reflects the addition of metabolically targeted tumor visualization; the European cost-effectiveness data (13–15) and the Roder trial (28) jointly support this tier as the cost-effectiveness frontier of intraoperative imaging.

**Tier 3** adds high-volume specialist services. The minimum institutional requirement is a neuropathology service capable of supporting on-site molecular diagnostics, sufficient case volume to maintain operator proficiency across multiple modalities (typically ≥50 high-grade glioma cases per year), and infrastructure for advanced iUS modes including CEUS and intraoperative imaging-based neuronavigation update. Tier 3 is the appropriate setting for the introduction of SRH, where the FDA-cleared and CE-marked Invenio NIO platform may substitute for or supplement frozen-section neuropathology.

**Tier 4** adds iMRI, restricted to referral centers with very high case volumes (typically ≥150 cases per year), the capital infrastructure for installation (US$1–10 million depending on field strength), the supporting cryogen and shielding infrastructure, and the operative time tolerance to accommodate the additional ∼100 min per case demonstrated by the Roder trial (28). Tier 4 should be entered only after Tier 3 maturity, and its addition should be evaluated against the published evidence that iMRI provides no clinical superiority over 5-ALA on EOR, PFS, or OS endpoints (28, 47).

HSI and AR neuronavigation are deliberately placed outside the tier system as research-stage modalities awaiting IDEAL stage 3 evidence (58, 59). Their evaluation should be prioritized within the Global IDEAL Sub-Framework structure (55) at centers with the infrastructure to support such studies.

The pathway is designed to be entered at any tier and progressed sequentially. It is not a one-size-fits-all deployment plan, but rather, it is a decision scaffold for centers assessing where rational investment lies given current infrastructure, case volume, and regulatory environment.

## 6 Discussion

This mini review has argued that the existing literature on intraoperative imaging in brain tumor surgery treats the modalities as a hierarchy of technological sophistication, when re-examined under the constraints of finite capital, scarce specialist workforce, and regulatory diversity that obtain in most of the world's neurosurgical settings, the apparent hierarchy inverts. The cost-effectiveness data favor 5-ALA, iUS, and fluorescein over iMRI. The accessibility barriers are dominated by capital infrastructure and regulatory status rather than consumable cost, and the proposed four-tier staged adoption pathway operationalizes these findings into a deployable sequence. The article's core empirical anchors are the Roder trial demonstrating no clinical superiority of iMRI over 5-ALA (28), the Coburger three-way comparison demonstrating that iMRI's gadolinium-dependent imaging detects only 50% of invasive tumor at the resection border (29), the Pichardo-Rojas meta-analysis demonstrating substantive survival benefit of iUS (32), and the Brazilian compounded-5-ALA experience (19), and collectively they make the argument empirically defensible.

Several limitations of the evidence base deserve explicit acknowledgement (summarized in Table 3). The most significant is the absence of any published cost-effectiveness analysis from a low- or middle-income country payer perspective for any modality. There is no published randomized controlled trial of iUS in any setting. The Cochrane network meta-analysis identified this gap and the planned FUTURE-GB trial may eventually address it (45). There is no head-to-head randomized comparison of iMRI and iUS. For HSI, SRH, and AR, no cost-effectiveness data of any kind exist. The proposed staged adoption pathway is itself a hypothesis requiring prospective evaluation. The Global IDEAL Sub-Framework (55) provides the methodology for that evaluation, and the WHO-INTEGRATE framework (57) provides the evidence-to-decision scaffold for adoption decisions made by individual centers or national systems.

**Table 3:** Evidence gaps and proposed research priorities.

Evidence gap	Why it matters	Research approach
No published cost-effectiveness analysis adopts an LMIC payer perspective for any modality	All published economic appraisals use high-income country viewpoints; no analysis applies the country-level opportunity-cost thresholds derived by Woods et al. (46); none models catastrophic or impoverishing healthcare expenditure	Health-economic analyses with country-specific opportunity-cost thresholds; modeling of catastrophic expenditure following Lancet Commission on Global Surgery indicators (7)
No randomized controlled trial of iUS exists in any setting	iUS is the modality with the most favorable cost/QALY among hardware modalities (24); the FUTURE-GB trial (45) is the planned remedy	Multicenter RCT comparing iUS-guided vs conventional resection, ideally with LMIC participation under Global IDEAL Sub-Framework (55)
No head-to-head RCT of iMRI vs iUS exists	Direct comparison is essential for rational tier allocation; current comparisons are observational	Pragmatic RCT or large prospective registry stratified by tier eligibility
No cost-effectiveness data of any kind exist for HSI, SRH, or AR neuronavigation	Adoption decisions for the three emerging modalities are being made without economic appraisal	IDEAL stage 2b/3 studies with built-in health-economic evaluation following established reporting standards
Single-technology HTAs absent for iMRI, iUS, HSI, SRH, and AR by major appraisal agencies (NICE, CADTH, IQWiG)	The absence prevents formal adoption recommendations and complicates national reimbursement decisions	Commissioning of single-technology appraisals by national HTA agencies
No prospective evaluation of any staged intraoperative-imaging adoption pathway exists in any setting	Implementation pathways have been proposed for medical devices in general (55) but not for the brain-tumor-imaging-specific case proposed here	Multicenter implementation study using NASSS framework (56) and WHO-INTEGRATE evidence-to-decision framework (57)

HTA, health technology assessment; LMIC, low- and middle-income country; NASSS, Non-adoption, Abandonment, Scale-up, Spread, Sustainability framework; RCT, randomized controlled trial; WHO, World Health Organization.

A further limitation concerns the place of intraoperative imaging within the wider chain of glioma care. Maximal safe resection is necessary but not sufficient for survival in high-grade glioma; outcome also depends on timely histopathological diagnosis, molecular characterization where available, and prompt access to radiotherapy and chemotherapy. In many resource-limited systems the principal bottlenecks lie outside the operating room, in delays in pathology reporting, absent molecular testing, and protracted waits for adjuvant therapy that may erode the survival advantage of improved resection. Under such conditions the marginal benefit of upgrading between imaging modalities may be smaller than that of strengthening pathology services or shortening time to adjuvant treatment. This does not weaken the case for prioritizing accessible and cost-effective imaging; it situates the staged adoption pathway as one component of a broader investment strategy, to be weighed against parallel needs across the diagnostic and therapeutic chain so that gains in resection are not lost downstream.

The closing claim is substantive. In the molecular era of glioma surgery, the imaging modalities that biologically delineate non-enhancing infiltrative disease, 5-ALA fluorescence, iUS with contrast-enhanced ultrasound, and the emerging Raman and stimulated Raman histology technologies, deserve preferential investment over modalities whose primary contribution is improved delineation of gadolinium-enhanced disease. Molinaro et al.'s demonstration that maximal resection of both contrast-enhanced and non-contrast-enhanced tumor in younger patients with IDH wild-type glioblastoma can approximate the survival of the favorable IDH mutant subtype (4), and Karschnia et al.'s confirmation that extensive resection benefits glioblastoma without contrast enhancement (6), together raise the imaging bar beyond what gadolinium can show. iMRI's gadolinium-dependent visualization is, on this argument, the wrong tool for the modern surgical target. For centers in resource-limited neurosurgical settings, the absence of iMRI is not the gap that needs to be closed first. The gap that needs to be closed first is the absence of any intraoperative imaging at all, and the modalities that close that gap most cost-effectively, most accessibly, and with the best alignment to the molecular-era resection target are iUS and fluorescence-guided surgery. Realizing this potential at scale will require coordinated effort. Industry must offer price adjustment, regulatory expansion, and training partnerships, and healthcare systems in low- and middle-income countries must develop procurement strategies, workforce development, and infrastructure investment.
